# Dual roles of DDR1 in stromal remodeling and immune escape: a regulator of the tumor immune microenvironment

**DOI:** 10.3389/fimmu.2026.1941532

**Published:** 2026-09-18

**Authors:** Xiaoxiao Xu, Zhenxing Wang

**Affiliations:** 1 International Medical Services, Cancer Hospital of Dalian University of Technology, Cancer Hospital of China Medical University, Liaoning Cancer Hospital & Institute, Shenyang, China; 2 Department of Hematology and Breast Cancer, Cancer Hospital of Dalian University of Technology, Cancer Hospital of China Medical University, Liaoning Cancer Hospital & Institute, Shenyang, China

**Keywords:** cancer-associated fibroblasts, collagen remodeling, DDR1, immune exclusion, immunotherapy, tumor immune microenvironment

## Abstract

The discoidin domain receptor 1 (DDR1) is a collagen-activated receptor tyrosine kinase that orchestrates multiple cellular processes and remodels the tumor immune microenvironment (TIME). Notably, DDR1 promotes immune exclusion predominantly through its extracellular domain, which regulates collagen fiber alignment to form a physical barrier that impedes cytotoxic T cell infiltration, a function independent of its kinase activity. Moreover, DDR1 mediates cancer cell adhesion, migration, and metabolic reprogramming, while modulating cancer-associated fibroblasts, macrophages, and neutrophils to establish an immunosuppressive niche. Recently, DDR1-targeting strategies have been in preclinical and early clinical development, including selective small-molecule inhibitors, monoclonal antibodies, antibody–drug conjugates, and proteolysis-targeting chimeras. Combining DDR1 inhibition with immune checkpoint blockade has demonstrated synergistic antitumor efficacy in preclinical models. This review synthesizes the current understanding of DDR1 as a central regulator of the TIME and discusses the translational potential and challenges of DDR1-targeting strategies to enhance cancer immunotherapy.

## 1 Introduction

The tumor immune microenvironment (TIME) is a dynamic ecosystem comprising heterogeneous cellular populations, including tumor, stromal, and immune cells, embedded within a remodeled extracellular matrix (ECM) and secreted signaling factors (1). This interconnection of cellular and acellular components, facilitating immune evasion and maintaining immunosuppressive niches, critically governs tumor progression and therapeutic response (2). Consequently, novel therapeutic strategies have evolved beyond solely targeting tumor cells to include the systemic reprogramming of the immunosuppressive microenvironment. Within this framework, the discoidin domain receptor 1 (DDR1) has emerged as a critical player, mediating cell-microenvironment communication.

DDR1 is a unique receptor tyrosine kinase (RTK) activated by collagen, a major component of the extracellular matrix (ECM) (3). It regulates fundamental cellular processes including differentiation, proliferation, adhesion and migration, while simultaneously modulating ECM architecture and tumor microenvironment (TME) composition (4). Recent advances have revealed that DDR1 exerts its protumorigenic functions through two conceptually distinct mechanisms: kinase-dependent signaling cascades and kinase-independent scaffolding functions mediated by its extracellular domain (ECD) (4, 5). This functional duality is central to understanding DDR1’s complex and sometimes contradictory roles in cancer biology and immunotherapy response.

### 1.1 DDR1 splice isoforms: structural diversity and functional implications

The DDR family is single-span transmembrane proteins, consisting of extracellular N-terminal discoidin homology (DS) domains and DS-like domains, juxtamembrane domains and C-terminal tyrosine kinase domains. DDR1 exists as five splice variants generated by alternative splicing, including DDR1a, DDR1b, DDR1c, DDR1d, and DDR1e. Among these, DDR1a, DDR1b, and DDR1c encode full-length receptors containing the intracellular kinase domain, whereas DDR1d and DDR1e are truncated variants without kinase activity.

DDR1 binds collagens I–IV and VIII through its DS domain. Unlike classical RTKs that are rapidly activated by growth factors, DDR1 exhibits slow and sustained autophosphorylation occurring over minutes to hours. The juxtamembrane tyrosine Y513 and activation-loop residues Y792 and Y796 represent the principal autophosphorylation sites (6). The receptor’s extracellular region is cleaved by matrix metalloproteinases (MMPs), shedding soluble ectodomain fragments that modulate collagen organization and immune cell infiltration in the extracellular space. In addition, DDR1 undergoes ligand-induced internalization and degradation via both clathrin-mediated and caveolin-mediated pathways (7).

DDR1a and DDR1b are the most widely distributed isoforms. Studies have demonstrated that cells expressing DDR1b exhibit superior potency in adhesion and migration than DDR1a-expressing cells on both DDR1-selective substrata and collagen I. Mechanistically, this difference arises from the higher affinity of DDR1b for talin, a key cytoskeletal adaptor protein that links integrins to the actin cytoskeleton. Notably, both DDR1a and DDR1b are phosphorylated upon collagen stimulation, but the downstream signalings differ substantially due to their differential adaptor protein recruitment (8).

Overexpression of either DDR1a or DDR1b increases cell adhesion, although isoform-specific roles in cell attachment have been characterized. DDR1a facilitates leukocyte migration in three-dimensional collagen matrices by promoting the amoeboid movement of activated T cells and other leukocytes through collagen-rich tissues without enhancing firm adhesion to collagen (9). In macrophages, the DDR1b isoform specifically mediates collagen-induced differentiation and chemokine production via the p38 MAPK and NF-κB pathways (10). This isoform-specific signaling has direct implications for immune regulation, as differential expression of DDR1 isoforms may shift the balance between ECD-dependent scaffolding and kinase-dependent signaling. Therefore, a thorough understanding of isoform-specific functions is essential for developing targeted therapeutic strategies and predicting patient responses to DDR1-directed therapies.

### 1.2 Scope and distinction from previous reviews

Unlike previous reviews that have focused predominantly on discrete DDR1 biology in cancer, such as DDR1 kinase signaling (4), medicinal chemistry (11), tumor immunology (12), tumor progression and chemotherapy resistance (13), or patent landscape of DDR1 modulators (14). The present review offers a unique and integrated contribution by providing: (1) a unified mechanistic framework that explicitly synthesizes kinase-dependent and kinase-independent DDR1 activities to resolve apparent contradictions in the literature; (2) a systematic compartment-specific analysis across tumor cells, stromal cells, and immune cells, formalized in a conceptual matrix with direct therapeutic implications; (3) a biology-aligned translational framework that matches therapeutic modalities, including kinase inhibitors, ECD-targeted antibodies, PROTAC degraders, and ADCs, to the specific DDR1 function being targeted.

### 1.3 A unifying mechanistic framework for DDR1 dual roles

To provide conceptual synthesis rather than a conventional literature summary, we propose a unifying framework based on four axes: (1) catalytic versus non-catalytic DDR1 functions; (2) cell-associated versus shed DDR1-ECD; (3) tumor-cell versus stromal-cell versus immune-cell DDR1, and (4) collagen abundance, architecture, cleavage state, and subtype. Throughout the manuscript, we explicitly assign each discussed mechanism to this framework, enabling readers to appreciate how the same receptor can produce divergent outcomes depending on context.

## 2 Orchestrating stromal remodeling

The ECM, a major component of the TME, is the interstitial framework within the tissues, which plays vital roles in structural support, cell adhesion, storage of water and growth factors, and also regulating intracellular signaling pathways (5). The dysregulation of the ECM is a hallmark of cancer. During tumor progression, cancer cells induce ECM stiffening, which in turn promotes further malignant transformation. Additionally, the stromal extracellular matrix (ECM) shields cancer cell clusters by forming a dense collagen-rich physical barrier that prevents immune cell infiltration into the tumor core, a phenomenon defined as physical immune exclusion. This barrier generates an immunologically “cold” tumor microenvironment characterized by sparse T cell infiltration and poor response to immune checkpoint blockade, highlighting the critical role of ECM remodeling in immune evasion.

### 2.1 Tumor cell DDR1 regulating collagen deposition and alignment

The DDR1-ECD, but not the intracellular kinase domain, can modulate collagen fibrillogenesis by reducing the rate and quantity of collagen deposition, significantly changing fiber morphology and matrix mineralization (15). Sun et al. seminally demonstrated that the DDR1-ECD mediated collagen remodeling to promote the intratumoral penetration of immune cells and drive immune exclusion in triple-negative breast cancer (TNBC) (16). The binding of DDR1-ECD to collagen promotes aligned collagen fibres, building a fence around tumors that physically blocks CD8^+^ T-cell infiltration.

Additionally, full-length intact DDR1 with kinase domain was also confirmed to promote tumor growth by suppressing infiltration of T cells (17, 18). The activation of the VIRMA-mediated TFAP2A/DDR1 axis was further shown to modulate collagen fiber alignment and anti-tumor immune cell infiltration in breast cancer (19). The binding to collagen triggers DDR1 activation and subsequent STAT1 upregulation, resulting in disrupting the linear architecture of collagen fibers in ECM. The reshaped type III collagen-rich ECM serves as a barrier to metastasis for disseminated tumor cell (20, 21).

Interestingly, cleaved and intact forms of collagen I exert opposing effects on pancreatic ductal adenocarcinoma (PDAC) progression through differential regulation of DDR1. Specifically, MMP-cleaved collagen I activates a DDR1–NF-κB–p62–NRF2 signaling axis, thereby promoting PDAC bioenergetics, macropinocytosis, tumor growth, and metastasis. In contrast, intact collagen I induces DDR1 degradation, which suppresses these oncogenic processes and restrains PDAC growth (21). This cleavage-dependent switch converts DDR1 from a tumor-suppressive to a tumor-promoting receptor, explaining why tumors with high MMP activity and collagen remodeling exhibit more aggressive behavior.

The conflicting outcomes of DDR1 ablation were demonstrated across different tumor types according to context-dependent dual-function models. In an immunocompetent KPC mouse model of PDAC, therapeutic blockade of DDR1 with DDR1-neutralizing antibody disrupted collagen architecture, enhancing CD8^+^ T cell infiltration (22). Notably, a pan-cancer analysis demonstrated DDR1 as a key mediator of collagen-driven immune resistance in multiple tumor types, including PDAC and other collagen-rich solid tumors. DDR1-mediated immune exclusion is a hypothesis that requires validation across cancer types, stromal architectures, and immunological contexts. Furthermore, a selective humanized DDR1 monoclonal antibody (mAb) PRTH-101 reversed immune exclusion and increased CD8^+^ T cell infiltration, an effect mediated through the disruption of collagen fiber alignment. Mechanistically, PRTH-101 inhibited DDR1 phosphorylation, which reduced collagen-mediated cell adhesion and significantly prevented the shedding of DDR1 from the cell surface (23). Furthermore, in an immunocompetent KRAS/p53-driven lung adenocarcinoma (LUAD) mouse model, inhibition and knockout of DDR1 promoted tumor growth with a decreasing of CD8^+^ T cells but an increasing of CD4^+^ helper T cells and regulatory T cells (24), suggests that the deletion of DDR1 also impacted the recruitment or proliferation of CD8^+^ cytotoxic T cells. Rather than representing a contradiction, this finding can be interpreted through the lens of cell-type specificity. In the LUAD model, DDR1 expressed in non-tumor compartments, such as fibroblasts or immune cells, may be required for optimal CD8^+^ T cell recruitment and maintenance of an anti-tumor immune milieu. Thus, the net effect of DDR1 ablation depends on the relative contributions of tumor cell DDR1 versus stromal or immune cell DDR1. This duality underscores that DDR1 functions are highly context-dependent, shaped by collagen matrix architecture, cell-type-specific expression patterns, and the dominance of kinase-dependent versus ECD-dependent signaling in distinct tumor subtypes. **Figure 1** illustrates the collaboration of DDR1 and collagen in tumor immunology.

**Figure 1:**
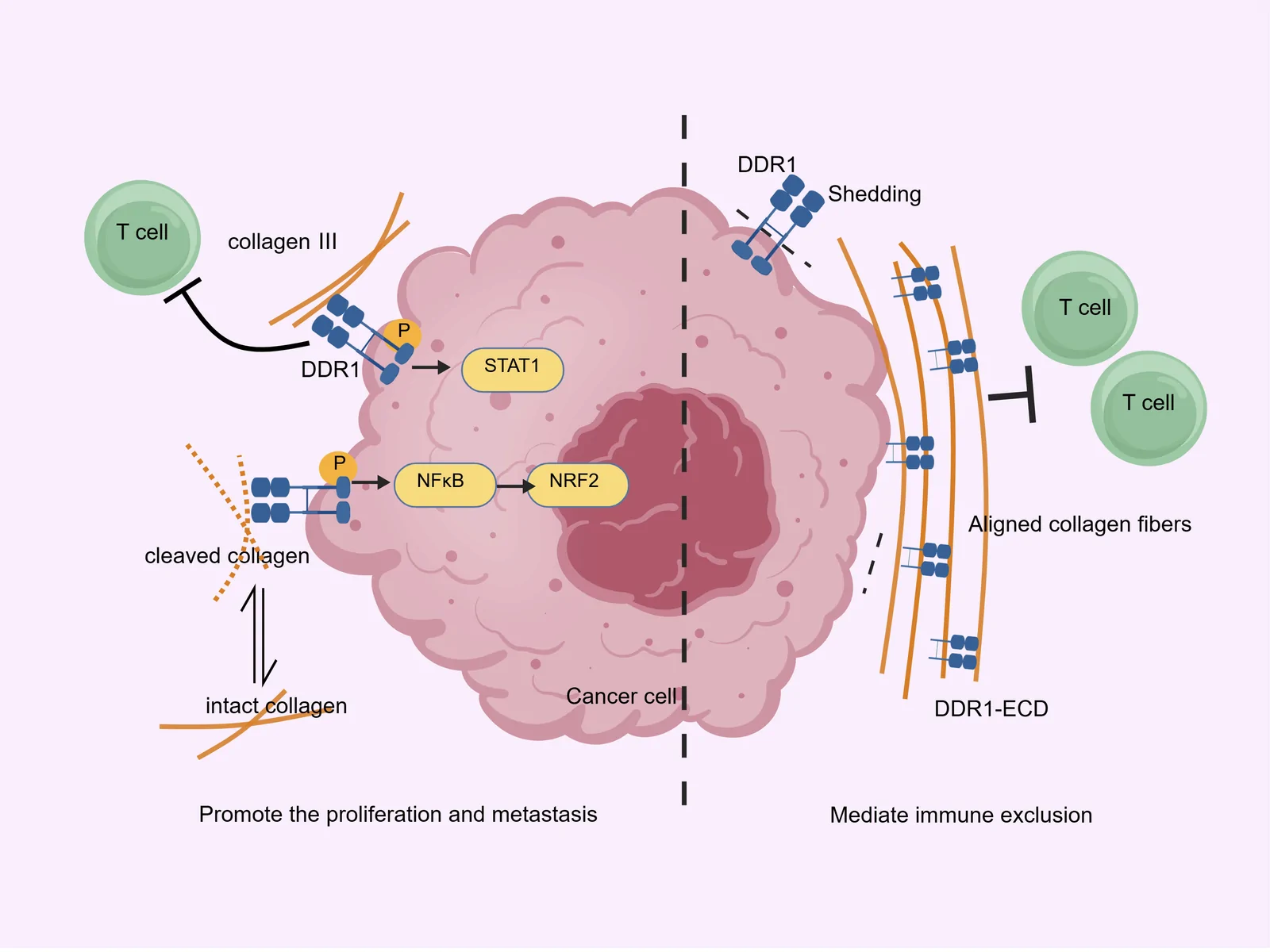
Collaboration of DDR1 and collagen in tumor immunology. (Left panel: cleaved collagen and DDR1 kinase signaling) DDR1 binds to MMP-cleaved collagen fragments, activating kinase-dependent signaling pathways that promote tumor proliferation, metabolic reprogramming, and metastasis. (Right panel: shed DDR1-ECD and collagen alignment) Shed DDR1 extracellular domain (DDR1-ECD) shed by MMPs promotes the alignment of collagen fibers into linearized, dense bundles, functioning as a physical barrier for immune cell exclusion.

### 2.2 Mediating cell migration and adhesion to ECM

The spreading and migration of cells on collagenous matrices are precisely regulated by the dynamic balance between contractile and protrusive forces, and the collagen receptor DDR1 critically regulates this process. DDR1 facilitates directional migration but paradoxically inhibits cell spreading, which is governed by its interaction with non-muscle myosin IIA (NMIIA) filament assembly on collagen. In stromal cells, high expression of DDR1 enhances calcium influx and potentiates non-muscle myosin IIA (NMIIA)-mediated contractility, thereby promoting collagen alignment and matrix compaction (25). Conversely, pharmacological inhibition of NMIIA increases spreading but impedes migration, indicating that DDR1 and myosin jointly govern motility by modulating adhesive contacts with collagen (26). Further studies have revealed that DDR1 functionally associates with ion channels like the transient receptor potential vanilloid type 4 (TRPV4) at collagen adhesion sites. The scaffold protein MRIP controls DDR1 clustering and activation, facilitating cell migration and tractional collagen remodeling by governing the DDR1-NMIIA interaction through a kinase-dependent mechanism (27). Beyond NMIIA, in BRAF-mutant melanoma cells, the focal adhesion protein kindlin-3 enhances oncogenic properties via collagen-activated DDR1 and its crosstalk with β1-integrin crosstalk, leading to decreased adhesion and increased migration (28).

### 2.3 Modulating activity and function of stromal cells

Stromal cells constitute a critical component of the TME. Accumulating evidence indicates that the tumor stroma plays an active role in modulating antitumor immune responses.

#### 2.3.1 Cancer-associated fibroblasts

Fibroblasts are a predominant cell type in the TME and critically regulate ECM formation and remodeling. They produce both structural components like collagen, fibronectin, and laminin and key enzymes for their modification and degradation such as lysyl hydroxylases and metalloproteinases (29). Fibroblasts are highly adaptable cells and are modulated by diverse signals, including cytokines, biochemical cues, and environmental stress. Within the tumor microenvironment, DDR1 serves as a pivotal regulator of CAFs activation and function.

Fibroblast heterogeneity has been extensively characterized by single-cell RNA sequencing, which has delineated multiple CAF subsets with distinct transcriptional signatures, including myofibroblastic CAFs (myCAFs), inflammatory CAFs (iCAFs), antigen-presenting CAFs (apCAFs) and vascular-associated CAFs (30). The differential expression of DDR1 and DDR2 across CAF subtypes has significant implications for therapeutic targeting. Single-cell RNA-sequencing analyses have demonstrated that both DDR1 and DDR2 are expressed in CAFs, but in distinct CAF subsets. DDR1 is predominantly expressed in apCAFs, while DDR2 is enriched in iCAFs (31). Notably, the specific DDR1 isoforms expressed in CAFs may differentially regulate collagen remodeling and CAF activation states, with DDR1b potentially playing a dominant role in mechanotransduction and matrix stiffness sensing (29). This differential expression pattern suggests that DDR1 may assume fundamentally distinct functional roles across CAF subtypes. Moreover, inhibition of DDR1 in tumor cells may be complemented by compensatory DDR2 signaling in CAFs, necessitating dual DDR1/DDR2 inhibition for complete disruption of stromal-mediated tumor promotion.

Functionally, DDR1 has been implicated in multiple aspects of CAF biology. Knockdown of DDR1 significantly reduces the expression of ACTA2, a hallmark of myCAFs, indicating that DDR1 is a critical determinant of myofibroblastic differentiation state (31). Furthermore, once activated, fibroblasts promote fibrosis by altering ECM secretion. Matrix stiffness-induced phosphorylation of DDR1 can suppress the synthesis of ECM proteins in fibroblasts through the TGF-β/STAT3 mechanotransduction axis (32). In addition, DDR1 modulates the migratory ability of fibroblasts within collagen matrices, which is relevant to CAF movement and infiltration in the tumor microenvironment (33). Collectively, these findings underscore the subtype-dependent roles of DDR1 in CAF heterogeneity and highlight its involvement in key functional attributes, including myofibroblastic differentiation, ECM remodeling, and cell migration.

#### 2.3.2 Hepatic stellate cells

The function of tumor DDR1 in the hepatic ECM remodeling was determined by the interaction with the hepatic sinusoidal cells (SCs), including sinusoidal endothelial cells, Kupffer cell, and HSCs (34, 35). SCs secretes soluble factors to induce DDR1 phosphorylation and MMP2 upregulation, facilitating stromal cell recruitment into the colorectal cancer (CRC) microenvironment. Furthermore, tumor-expressed DDR1 enhanced the capacity of SCs to modify the ECM organization by regulating collagen and MMP expression (36). In uveal melanoma, DDR1 and its ligand may mediate the interaction between tumor cells and their surrounding niche to promote the metastatic colonization in the liver. Mechanistically, cancer cells secrete TGF-β1 to activate HSCs, which increase the expression of collagen type I. In turn, the remodeled extracellular matrix stimulated DDR1-STAT3 pathway, resulting in strengthened survival, enhanced stemness and clonogenicity in cancer cells (37).

#### 2.3.3 Vascular smooth muscle cells

VSMCs are the predominant cell type in the vascular media and play essential roles in maintaining vessel tone, remodeling the vascular extracellular matrix, and responding to mechanical cues from the microenvironment. Evidence from non-cancer vascular pathology indicates DDR1 mediates VSMCs adhesion, migration, and proliferation on collagen, critically promoting neointimal thickening and collagen deposition after arterial damage in vascular injury (38). Inhibition of DDR1 disrupts both tumor vessel infiltration and injury-induced arterial remodeling, highlighting its broad therapeutic potential in diseases characterized by aberrant vascular responses. In addition, Lu et al. further showed that collagen-stimulated VSMC migration via DDR1 requires Src kinase activity and is associated with increased MMPs expression (39). Furthermore, DDR1 tyrosine kinase activity has been shown to play a central role in this process as a core mechanosensor and signal transducer, enabling VSMCs to sense matrix stiffness and initiate pathological calcification programs in non-cancer settings. When VSMCs are cultured on collagen I-coated substrates of varying stiffness, increased substrate rigidity upregulates both DDR1 expression and its phosphorylation, companied by enhanced nuclear localization of the Runt-related transcription factor 2 (Runx2), upregulation of other osteochondrogenic markers, and ultimately increased *in vitro* calcification (40). Another study further confirms that DDR1, activated by ECM stiffness, not only directly regulates pathological calcification but also drives VSMCs toward a pro-inflammatory phenotype via the DDR1-ERK-p53-DNMT1 signaling axis. This transformation increases inflammatory cytokines secretion, including monocyte chemoattractant protein-1 (MCP-1) and interleukin-6 (IL-6). These cytokines subsequently promote monocyte and macrophage recruitment into the vascular microenvironment, establishing a feed-forward loop wherein stiffness-induced DDR1 activation increases inflammation and myeloid cell infiltration (41).

The VSMC mechanosensing and pro-inflammatory functions observed in non-cancer settings may have relevance to the tumor microenvironment, where tumor-associated blood vessels are exposed to altered ECM stiffness, collagen remodeling, and inflammatory signals. In the tumor context, this mechanism may contribute to the recruitment of tumor-associated macrophages and myeloid-derived suppressor cells, thereby fostering an immunosuppressive niche.

#### 2.3.4 Tumor angiogenesis

Tumor angiogenesis represents a key hallmark of cancer. Indeed, tumor vasculature was considered as a vital pathological feature of cancer by supplying nutrients and oxygen required to progress and metastasize (42). The formation of new blood vessels from the existing vascular network can occur at multiple stages of tumor development. Compared to normal blood vessels, tumor vessels are characterized by increased permeability, irregular morphology, and functional impairment (43).

Accumulating evidence suggests that DDR1 plays a role in cancer progression by regulating tumor angiogenesis. However, the mechanisms are multifactorial and require careful distinction. First, tumor-cell-autonomous DDR1 effects on angiogenic factor secretion. In an orthotopic nude mouse model, DDR1-silenced implanted tumors exhibited a significant reduction in both angiogenesis and lymphangiogenesis, consequently leading to decreased lymph node and liver metastasis, with downregulated expression of vascular endothelial growth factor (VEGF)-A, VEGF-C, and platelet-derived growth factor-B (PDGF-B) (44). Second, human umbilical vein endothelial cells (HUVEC) significantly increased tubular structures formation, when cultured in conditioned medium from DDR1 overexpression renal cancer cells (45). Third, DDR1 in oral squamous cell carcinoma (OSCC) cells promotes angiolymphatic invasion. Inhibiting DDR1 kinase activity disrupts cell cohesion and reduces invasion into blood and lymphatic vessels in xenograft models (46). In response to VEGF, endothelial cells (ECs) must degrade the basement membrane and surrounding ECM to form new vessels (47). DDR1 serves as a key mediator linking tumor-derived signals to endothelial invasive behavior, facilitating tumor angiogenesis by enabling ECs to navigate through collagen-rich matrices (39). Whether these effects are kinase-dependent or structural, and whether they operate in immunocompetent versus immunodeficient models, remains to be fully elucidated. Furthermore, angiogenesis contributes to immune escape by promoting tumor growth, altering vessel permeability, and creating hypoxic niches that favor immunosuppressive cell infiltration, and these connections warrant further investigation.

### 2.4 Spatial and single-cell resolution of DDR1 expression in the TIME

Emerging single-cell RNA sequencing (scRNA-seq) and spatial transcriptomic studies have established the expression of DDR1 across distinct cellular compartments within the TIME. In breast cancer and PDAC, scRNA-seq analyses have confirmed that DDR1 is predominantly expressed in malignant ductal cells, with DDR1-high tumors associated with reduced cytotoxic CD8^+^ cytotoxic T cells and increased immunosuppressive Tregs populations (16, 22). In PDAC, CD8^+^ T cells infiltrating DDR1-high tumors exhibited transcriptomic signatures of impaired function, including downregulation of pathways involved in antigen presentation (21). Spatial transcriptomic profiling has further uncovered collagenolysis-dependent DDR1 activation in specific tumor niches, highlighting the spatial heterogeneity of DDR1 signaling.

Beyond malignant cells, scRNA-seq has also revealed DDR1 expression in tumor-associated macrophages and neutrophils in non-small cell lung cancer (NSCLC). This cell-type-restricted expression has important functional implications, because DDR1 in tumor cells primarily promotes ECM remodeling and immune exclusion, while DDR1 in stromal or immune cells may have distinct, even opposing, effects on anti-tumor immunity. For instance, DDR1 expression in CAFs may contribute to matrix stiffness and immunosuppression (32), while DDR1 in macrophages directly regulates their polarization state (48). These compartment-specific functions underscore the importance of spatially resolved analyses for understanding DDR1 biology and predicting therapeutic responses to DDR1-targeted agents.

## 3 Directing immune escape

### 3.1 Regulation of T-cell function

Primary tumors are mainly composed of effector T cells, which express high levels of chemokine receptors and cytotoxic genes, with few exhausted T cells present. As the disease progresses to metastasis, TME induces progressive T cell dysfunction by increasing expression of co-inhibitory receptors, such as CTLA-4 and PD-1 (49). DDR1 suppresses anti-tumor immunity through both physical exclusion and directly functional regulating of immune cells (**Figure 2**).

**Figure 2:**
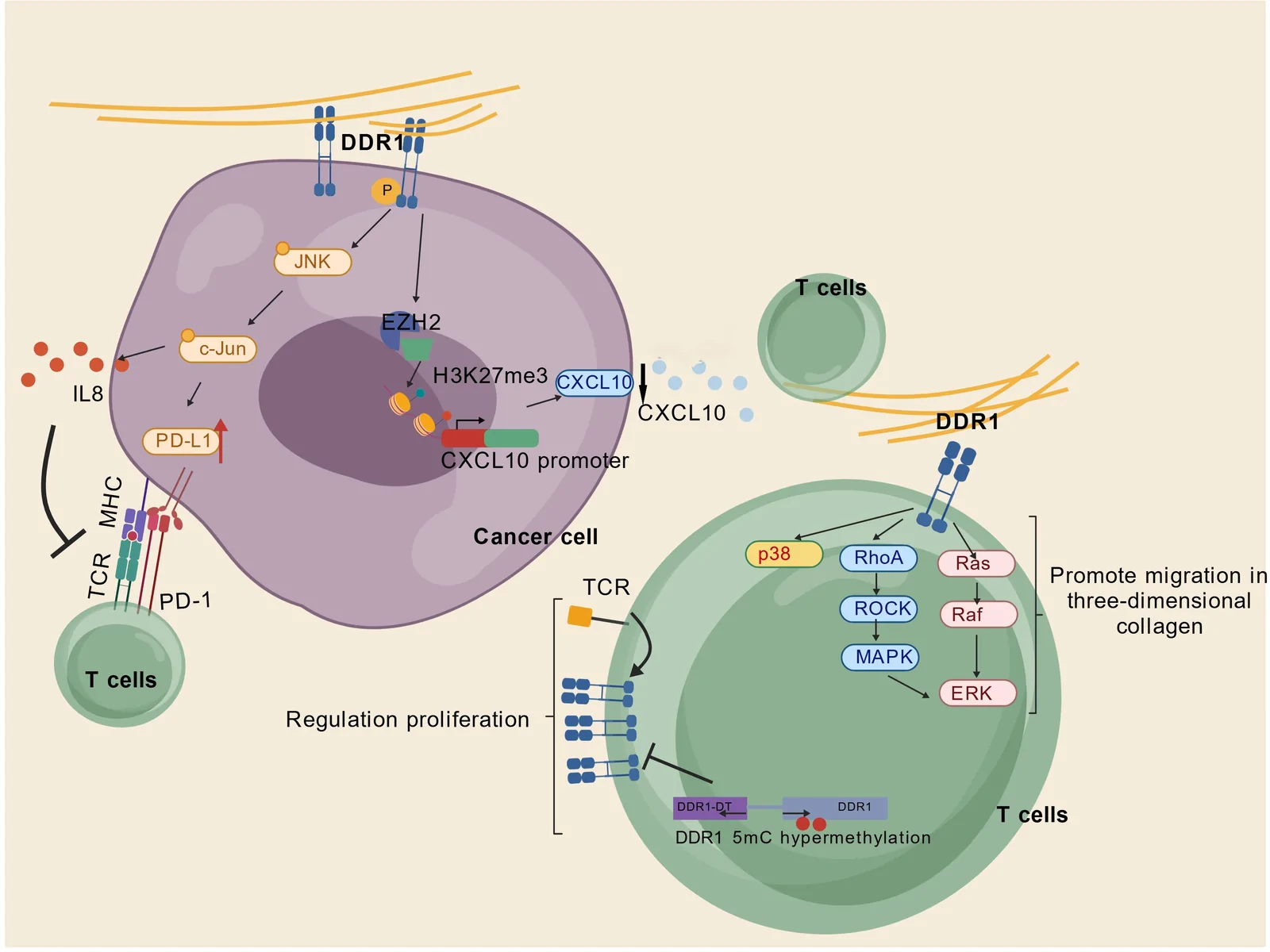
Mechanism and effects of DDR1 in regulating T cells. In cancer cells, DDR1 activation inhibits T cells function by releasing immunosuppressive cytokines and upregulating the expression of PD-L1. Concurrently, DDR1 in T cells promotes the migration and proliferation of T cells.

#### 3.1.1 DDR1 in T cells as a direct axis for migration and activation

Activation of primary human T cells with anti-CD3 mAb induced the expression of DDR1. DDR1 promotes the migration of activated T cells in three-dimensional (3D) collagen. Furthermore, overexpression of DDR1 in the monocytic cell line THP-1 was reported to enhance their migration in 3D collagen (9). Mechanistically, such TCR-induced DDR1 promotes amoeboid movement of activated T cells in collagen matrices through the Ras/Raf/ERK MAP Kinase and PKC cascades, without enhancing adhesion to collagen (50, 51). In addition, DDR1 is expressed in the vast majority of human Th17 cells and promotes Th17 migration via the activation of RhoA/ROCK/MAPK/ERK signaling, a key pathway enabling effector T cells to migrate through collagen of perivascular tissues (52). In another study, DDR1 promotes Th17 migration in three-dimensional collagen by p38 activation, which is implicated in T cell activation, immune and inflammatory responses (53). Beyond the signaling pathways that regulate DDR1 expression in T cells, leukocyte DDR1 hypermethylation was described in a strong positive correlation with neutrophil counts and a negative correlation with lymphocyte counts. The underlying mechanism may involve impaired lymphocyte proliferation, as promoter hypermethylation prevents the binding of PAX5, a transcription factor for B-cell lineage differentiation, to the regulatory region of DDR1 (54).

#### 3.1.2 Tumor cell DDR1-mediated T cell suppression

DDR1 in tumor suppresses T cell migration by regulating chemokine expression. Critically, DDR1 suppresses CXCL10 transcription via upregulating EZH2-mediated H3K27me3 at the CXCL10 promoter. This suppression of CXCL10, a potent T cell chemoattractant, reduces CD8^+^ T cell infiltration. Accordingly, DDR1 knockdown or inhibition relieves CXCL10 suppression and increases CD8^+^ T cell infiltration in microsatellite stable (MSS) CRC (55). In addition, DDR1 is identified as a negative immunomodulator in CRC by inhibiting IL-18 synthesis, leading to low infiltration of CD4^+^ and CD8^+^ T cells (56). Furthermore, DDR1 enhances the expression of PD-L1 via activating the c-Jun N-terminal kinase (JNK) signaling pathway, which might also contribute to immune evasion. A bioinformatics analysis in cervical cancer shown the role of DDR1 in immune escape. Specifically, elevated DDR1 expression correlated with an enrichment of resting memory CD4^+^ T cells while low DDR1 levels were linked to greater cytotoxic CD8^+^ T cell infiltration. Analysis of immune checkpoint revealed that high DDR1 expression was associated with decreased LAG3 but elevated CD47 levels. Further analysis of molecular functions suggesting its involvement in remodeling the tumor microenvironment and associated metabolic reprogramming (57).

Above all, these findings establish DDR1 as an intrinsic regulator of T cell motility, suggesting that DDR1-targeted therapies could directly modulate T cell trafficking independently of ECM remodeling. This immune cell-intrinsic function of DDR1 must be carefully considered in therapeutic strategies, as DDR1 inhibition in T cells might impair effector T cell migration into tumors, counteracting the benefits of relieving ECM-mediated physical exclusion.

### 3.2 Direct regulation of immunosuppressive cell function

#### 3.2.1 Recruiting and differentiation of macrophages

Macrophages play vital roles in both innate and adaptive immunity, capable of phagocytosis and antigen presentation (58). Notably, DDR1 is expressed during the process of human monocytes differentiation into macrophages. During inflammatory responses, the activation of DDR1b isoform with collagen facilitated their differentiation and increased the production of cytokine/chemokine, including IL-8, macrophage inflammatory protein-1alpha (MIP-1α), and MCP-1, in human macrophages via the p38 mitogen-activated protein kinase (MAPK) and NF-kappaB-dependent manner (10). Furthermore, abnormal DDR1 promotes collagen-induced activation and function of macrophages. Activation of DDR1 with collagen in murine macrophage cell line induces inducible NO synthase (iNOS) and Nitric oxide (NO) production, which acts as important regulators of immune-inflammatory responses, through activating NF-kappaB, p38 MAPK, and JNK (59). Additionally, inhibition of DDR1 effectively inhibited macrophage migration and the production of inflammatory cytokines, such as NO, iNOS, COX-2, IL-6 and TNF-α (60, 61). During atherogenesis, DDR1 promotes macrophages infiltration and recruitment. Notably, *in vitro* studies revealed that macrophages from Ddr1(-/-) mice decreased adhesion and chemotactic invasion of type IV collagen with reduced expression of MCP-1 and vascular cell adhesion molecule-1 (62, 63).

Macrophages can differentiate into tumor-associated macrophages (TAMs) after migrating into the TIME. In response to various microenvironmental stimuli, macrophages can differentiate into specific pro-inflammatory macrophages (M1-like) or anti-inflammatory macrophages (M2-like) phenotypes with specific biological functions (64, 65). The binary M1/M2 classification oversimplifies TAM heterogeneity in human tumors, as modern immunology recognizes multiple context-dependent states not captured by this dichotomy. We therefore use “M1-like” and “M2-like” when referring to experimental marker panels and acknowledge the limitations of this classification. Studies have demonstrated that DDR1 promotes the polarization of TAMs to the immunosuppressive M2-like phenotype, thereby shaping an immune-tolerant tumor microenvironment that facilitates tumor progression and immune evasion (16). Specifically, tumor-cell DDR1 induces p-c-Jun-dependent IL33 transcription to drive M2-like macrophage polarization, establishing a DDR1-DDX21-c-Jun-IL-33 axis that drives immunosuppression in colorectal cancer (48). In gastric cancer, the expression of DDR1 is negatively associated with monocytes, TAMs, and M2-like macrophages, indicating its involvement in regulating macrophage polarization (66). Furthermore, DDR1 activation in macrophages is proved to regulated NLRP3 inflammasome and macrophage responses, including macrophage activation, and M1/M2 polarization in idiopathic pulmonary fibrosis (IPF) (67).

Thus, DDR1 is therapeutically targetable, representing a direct immunostimulatory axis. DDR1 blockade in TAMs can repress M2-like polarization and promote an anti-tumor M1-like phenotype independent of its effects on ECM remodeling. Importantly, DDR1-targeted therapies may exert dual benefits, relieving physical barriers via ECD blockade and directly reprogramming TAMs toward an anti-tumor phenotype. Distinguishing between tumor cell DDR1 and immune cell DDR1 effects is essential for optimizing combination strategies with immune checkpoint inhibitors.

#### 3.2.2 Driving neutrophil extracellular trap formation

Neutrophils are important components of tumor immune microenvironment (TIME) and play a key role in tumor progression and therapy resistance (68). Based on its interaction with collagen receptors, DDR1 facilitates the adhesion, migration, differentiation, maturation, and cytokine/chemokine production of human leukocytes, including neutrophils, monocytes, and lymphocytes (69). Specifically, studies have linked neutrophil extracellular traps (NETs), released by activated neutrophils to cancer initiation and progression (70, 71). NETs consist of decondensed chromatin filaments coated with various granular proteins including neutrophil elastase, cathepsin G, myeloperoxidase, MMP-9 and histones (72). They may also directly stimulate tumor growth via proteases and trap circulating tumor cells as an adhesive scaffold to enhance their attachment, invasion, and migration.

Recent studies have confirmed that tumor-cell-DDR1, rather than neutrophil-intrinsic DDR1, mediated NET formation. In PDAC cancer cells, collagen-induced DDR1 activation in PDAC cancer cells increases the production of CXCL5, promoting tumor-associated neutrophils recruitment and the formation of NETs, ultimately driving cancer cell invasion and metastasis by a DDR1/PKCθ/SYK/NF-kappaB signaling cascade. Specifically, DDR1 engagement by collagen activates PKCθ, which phosphorylates SYK, inducing NF-κB nuclear translocation and transcriptional upregulation of CXCL5. Then, secreted CXCL5 recruits neutrophils via CXCR2, promoting their activation and NET release. The NETs, enriched with neutrophil elastase and MMP-9, subsequently degrade the ECM and enhance tumor cell invasion and metastasis (73). This DDR1/CXCL5/NETs axis establishes a critical link between collagen-DDR1 interaction and the formation of an immune microenvironment that facilitates PDAC metastasis, highlighting a non-cell-autonomous role of DDR1 beyond its intrinsic effects on cancer cells. Similarly, collagen-activated DDR1/CXCL5, which in turn promotes NET formation, drives tumor growth and metastasis in breast cancer through facilitating the differentiation and immune infiltration of Tregs (74).

### 3.3 DDR1-mediated metabolic reprogramming in TIME

Distinct from normal cells, cancer cells undergo metabolic reprogramming to support heightened biosynthetic and energetic needs, another hallmark of cancer (75). These shifts in lactate, amino acid, and lipid metabolism not only drive tumor proliferation but also remodel the TME that impacts immune cell function, thereby promoting tumor progression (76). Recently, studies indicated that DDR1 participated in energy metabolic reprogramming to regulate cancer progression in various cancers. In CRC cells, DDR1 facilitated glucose reprogramming via PI3K/AKT/PKM2 signaling pathway, thereby supporting extracellular acidification and cell proliferation (64). In breast cancer, DDR1 regulates mitochondrial ATP production and metabolism-related proteins in both glycolysis and oxidative phosphorylation, including MCT1, MCT4, hexokinase 2, PKM2, NRF-1 (72, 77). Additionally, in head and neck squamous cell carcinoma (HNSCC), DDR1 sustains tumor cell survival by promoting monounsaturated fatty acid (MUFA) biosynthesis, thereby suppressing ferroptosis. Specifically, inhibition of DDR1 relieves MUFA-mediated ferroptosis suppression, and triggers ferroptosis-mediated immunogenic cell death, which in turn enhances CD8^+^ T cell infiltration and cytotoxicity, potentiating the antitumor efficacy of carbon ion radiotherapy (78). Therefore, DDR1 regulates the tumor’s aberrant metabolism on the environment and impacts the immune cells within it. Collectively, **Figure 3** summarizes multifaceted mechanisms of DDR1 in regulating pro-tumorigenic TIME.

**Figure 3:**
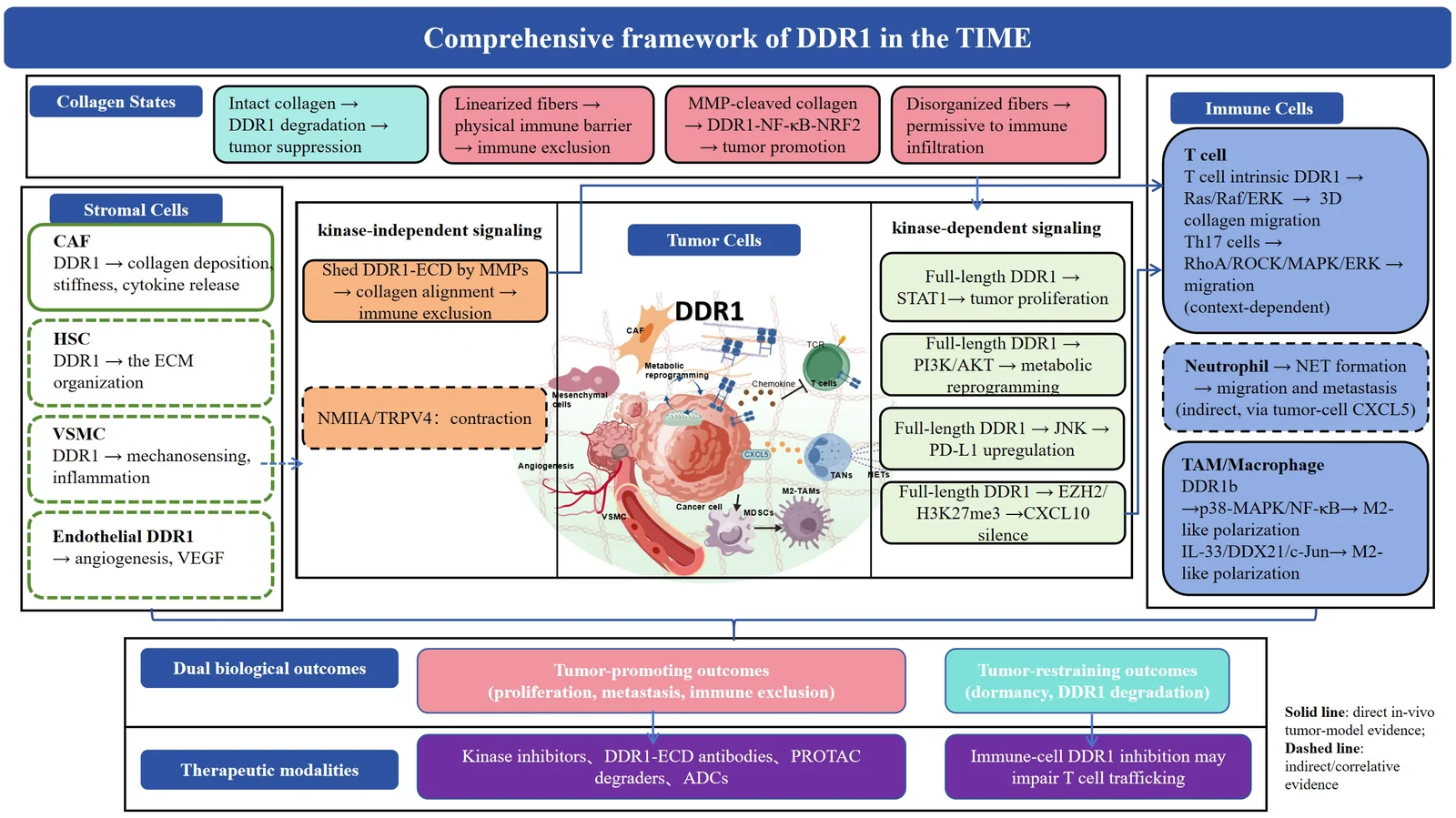
Multifaceted mechanisms of DDR1 within the tumor immune microenvironment (TIME). DDR1 exerts context‑dependent functions across multiple cellular compartments. Tumor‑cell DDR1 mediates kinase‑dependent intracellular signaling cascades and kinase‑independent extracellular‑domain (ECD) functions including MMP‑dependent shedding of soluble DDR1‑ECD, collagen fiber alignment and physical immune barrier formation via NMIIA/TRPV4. In stromal compartments, DDR1 modulates cancer‑associated fibroblasts (CAFs), vascular smooth muscle cells (VSMCs), endothelial cells and hepatic stellate cells (HSCs) to drive ECM remodeling, mechanosensing, pro‑inflammatory cytokine secretion and angiogenesis. Within immune compartments, cell‑intrinsic DDR1 regulates T‑cell 3D‑collagen migration, promotes M2‑like polarization of tumor‑associated macrophages (TAMs); tumor‑cell‑derived CXCL5, downstream of DDR1, recruits neutrophils and triggers NET formation to support metastasis. Divergent collagen status determines whether DDR1 drives tumor‑restraining versus tumor‑promoting outputs. Created with BioGDP.

## 4 Clinical translation of DDR1-targeted therapy

### 4.1 Therapeutic approaches targeting DDR1

Increasing evidence have demonstrated the potential roles of DDR1 in regulation of cellular morphogenesis, differentiation, proliferation, adhesion, migration, and invasion across various solid tumors (79–81). Additionally, DDR1 was found to instigate immune exclusion (16, 23). Therefore, the multifaceted role of DDR1 in solid tumors makes it a promising new drug target. To date, various DDR1-targeted therapies have been developed for the treatment of cancer. Unfortunately, the present medications that target DDRs have moderate to high toxicity and insufficient selectivity, which leads to suboptimal therapeutic and off-target consequences. Despite the development of several DDR1-targeted inhibitors for oncological applications, no selective small-molecule inhibitor has advanced to oncology clinical trials.

#### 4.1.1 Small-molecule DDR1 inhibitors

Small-molecule DDR1 inhibitors are the most extensively studied therapeutic approach. By binding to the intracellular kinase domain (KD) region, these inhibitors exert antitumor effects by inhibiting the phosphorylation of DDR1 and kinase activity and hindering downstream signaling.

Given the high structural homology between the KD region of DDR1 and those of other RTKs, certain RTK inhibitors have demonstrated poorly selective inhibitory activity against DDR1 but contributes to systemic toxicity. For instance, Breakpoint Cluster region- Abelson (Bcr-Abl) multitarget inhibitors, including Nilotinib, Imatinib, and Dasatinib, have shown inhibitory effects on collagen-induced autophosphorylation of DDR1 and off target toxicity (82–84). Here, we focus on selective DDR1 inhibitors on the field of tumor therapy (**Table 1**). Numerous selective DDR1 small-molecule inhibitors exhibited favorable pharmacokinetic properties and antitumor efficacy in preclinical models.

**Table 1:** DDR1-targeted therapies on tumor cells.

Modality	Drug name	Molecular mechanism	Kinase dependence	Assay type & DDR1 potency	Cancer indication	*In vivo* evidence	Stage	Key limitation
Small-molecule kinase inhibitor	compound 1 (DDR1-IN-1) (106)	Kinase inhibition	Kinase-dependent	Biochemical IC_50_ = 105 nM	CRC, BC, NSCLC, sarcoma, HCC	Yes (xenograft)	preclinical	Limited selectivity; no DDR2 data
	DDR1-IN-2 (106)	Kinase inhibition	Kinase-dependent	Biochemical IC_50_ = 47 nM	CRC, BC, NSCLC, sarcoma, HCC	Yes (xenograft)	preclinical	Limited selectivity; no DDR2 data
	6c (107)	Kinase inhibition	Kinase-dependent	Biochemical IC_50_ = 44 nM	BC, CRC,	No	preclinical	No *in vivo* data
	7rh (108, 109)	Kinase inhibition	Kinase-dependent	Biochemical IC_50_ = 6.8 nM	NSCLC, BC, CRC, CML, GC, NPC	Yes (xenograft)	preclinical	Limited selectivity
	7rj (108, 109)	Kinase inhibition	Kinase-dependent	Biochemical IC_50_ = 7.0 nM	NSCLC, BC, CRC, CML,	Yes (xenograft)	preclinical	Limited selectivity
	7f (110)	Kinase inhibition	Kinase-dependent	Biochemical IC_50_ = 14.9 nM	pancreatic cancer	Yes (xenograft)	preclinical	Limited selectivity
	8v (111)	Kinase inhibition	Kinase-dependent	Biochemical IC_50_ = 23.8 nM	NSCLC	No	Preclinical	No *in vivo* data
ECD-targeting	PRTH-101 (23, 112)	ECD neutralization (blocks collagen binding)	Kinase-independent	Cellular IC_50_ (pDDR1) = 61.4 ng/mL	advanced or metastatic solid tumors	Yes (breast cancer models)	Phase I clinical*; recruiting	No clinical results reported
Antibody–drug conjugates	DDR1-DX8951 (96)	ECD targeting + cytotoxic payload	Both	Cellular cytotoxicity IC_50_ = 0.067 nM	TNBC	Yes (TNBC models)	preclinical	Requires DDR1 expression
	T4H11-DM4 (97)	ECD targeting + cytotoxic payload	Both	Cellular cytotoxicity IC_50_ = 2.5 nM (HT-29); 22.1 nM (HCT116)	colon carcinoma	Yes (xenograft)	preclinical	Requires DDR1 expression
DDR1 degradation	Compound DP 1 (100)	Protein degradation (PROTAC, UPS-mediated)	Both	Cellular degradation DC_50_ = 0.73 nM	BC	Yes (breast cancer models)	preclinical	PK optimization needed
	LLC355 (101)	Protein degradation (autophagy-tethering, lysosomal-mediated)	Both	Cellular degradation DC_50_ = 150.8 nM	NSCLC	No	preclinical	No *in vivo* data

*NCT05753722: A First-in-Human Study of PRTH-101 Monotherapy ± Pembrolizumab in Subjects with Advanced Malignancies. Phase I, recruiting; no results reported as of August 2026.

Notably, a dual-target small-molecule inhibitor has been developed to concurrently disrupt DDR1 and the epidermal growth factor receptor (EGFR). This agent demonstrates a synergistic inhibitory effect, underscoring the potential therapeutic utility of co-targeting DDR1 and EGFR in DDR1/EGFR-positive NSCLC (85).

DDR2 exhibit distinct but complementary tissue-specific expression compared to DDR1, with DDR1 predominantly in epithelial cells and DDR2 in mesenchymal cells. Moreover, the distinction between DDR1 and DDR2 is their collagen specificity, with DDR1 being activated by both fibrillar and non-fibrillar collagens, whereas DDR2 activation is restricted to fibrillar collagens alone (7). These receptors engage in reciprocal signaling crosstalk, wherein DDR2 activation by fibrillar collagens induce DDR1 expression and transactivates DDR1 in stromal fibroblasts (86, 87). DDR2 may contribute to the establishment of an immunosuppressive tumor microenvironment through the recruitment of myeloid-derived suppressor cells (MDSCs) and the promotion of M2-like polarization of macrophages (88). Importantly, DDR1 and DDR2 can physically interact, and their co-expression has been shown to exert inhibitory effects on cell migration (89). A major limitation of selective DDR1 inhibitors is the potential for DDR2 upregulation to compensate for DDR1 inhibition. In CAF-rich tumors, DDR2-mediated signaling may sustain collagen-driven malignancy even when DDR1 is effectively blocked, representing a key resistance mechanism to monotherapy DDR1 inhibitors (90). Indeed, DDR2 may participate in establishing an immunosuppressive tumor microenvironment by recruiting myeloid-derived suppressor cells (MDSCs) and promoting M2 polarization of macrophages (91, 92). Consequently, this functional interdependence and compensatory signaling between the two receptors underscore the therapeutic rationale for developing dual DDR1/DDR2 inhibitors to fully disrupt collagen-driven malignancy.

#### 4.1.2 Antibody drugs

Monoclonal antibodies (mAbs) represent another promising class of DDR1 inhibitors, which target the extracellular discoidin domain or discoidin-like domain domains, block collagen binding and thereby inhibit DDR1 autophosphorylation. By recognizing unique epitopes not shared with other RTKs, mAbs overcome toxicity limitations with enhanced selectivity.

DDR1 promotes immune exclusion by aligning collagen fibers within the extracellular matrix. A neutralizing antibody targeting the DDR1-ECD was demonstrated to disrupt collagen alignment, enhance immune cell infiltration, and suppress tumor growth in immunocompetent breast cancer models. Subsequently, a novel humanized DDR1 antibody PRTH-101, which significantly blocked DDR1 shedding from the cell surface, alleviated immune exclusion, enhanced immune infiltration, and suppressed tumor growth in preclinical breast cancer models. Supported by these findings, PRTH-101 has advanced to phase I clinical trial (NCT05753722), which is recruiting and no results have been posted (23).

The anti-DDR1 mAb, competitively binding to DDR1 and its ligand collagen 1, synergized with anti-PD-1 therapy by employing dual immunomodulatory effects. It directly engages immune effector mechanisms via ADCC/CDC while also upregulating the chemokines CCL3 and CXCL11 to enhance CD8^+^ T cell recruitment and cytotoxic function (93). Consequently, DDR1 inhibition represents a multi-faceted approach that simultaneously disrupts oncogenic signaling and remodels immunologically “cold” tumors into “hot” ones, providing a strong rationale for its combination with PD-1 blockade in gastric cancer treatment.

#### 4.1.3 Antibody-drug conjugates

Antibody-drug conjugates (ADCs) are engineered targeted therapeutics consisting of a target-specific antibody linked to a potent cytotoxic payload, thereby combining the precision of targeted therapy with the potency of chemotherapy (94, 95). Moreover, beyond direct payload delivery, ADCs can also exhibit antibody-mediated immune effector functions and, in some cases, a bystander killing effect that extends their antitumor activity to neighboring cells.

DDR1-DX8951 is an ADC composed of an anti-DDR1 monoclonal antibody linked to the cytotoxic agent DX-8951 via a cleavable linker. In triple-negative breast cancer (TNBC) mouse models, the DDR1-DX8951 increased the percentage of tumor-infiltrated CD8^+^ T cells. Additionally, the combination of DDR1-DX8951 with pembrolizumab not only significantly inhibited tumor growth and metastasis, but also remodeled the immunosuppressive tumor microenvironment (TME) of TNBC, by elevating the infiltration of CD8^+^ T cells and serum levels of tumor-suppressing cytokines (TNF-α, IL-2, and IFN-γ) (96). Another anti-DDR1 ADC, T4H11-DM4, carrying the tubulin inhibitor payload DM4, showed antitumor effects and its safety in preclinical mouse models of colon carcinoma (97)and breast cancer (98).

#### 4.1.4 Protein degradation

Degradation of DDR1 represents a promising strategy due to the critical roles of the non-catalytic functions of DDR1 in tumor progression and immune exclusion. PROTAC (Proteolysis-Targeting Chimera) technology exploits the intracellular ubiquitin proteasome system (UPS) to selectively degrade the entire target proteins (99). A PROTAC molecule consists of three elements: a ligand that recruits an E3 ubiquitin ligase, a small-molecule warhead that binds to the target protein, and a chemical linker connecting the two. Notably, PROTACs not only block catalytic signaling but also ablate the non-catalytic scaffolding functions of inactive kinases, thereby inducing a profound and durable suppression of kinase activity. Moreover, PROTACs can achieve sustained target suppression at lower concentrations, potentially reducing off-target effects. Selective degradation of DDR1 by PROTAC approach have also been explored to exhibit efficacious anti-tumor activity and reverse immune exclusion for tumor immunotherapy. Compound DP 1, a degrader of DDR1 based on PROTAC strategy, demonstrated potent and nanomolar-range degradation potency against DDR1 across multiple cell lines. Notably, *in vivo* administration of DP1 effectively promoted the infiltration of CD8^+^ cells into the tumor core (100). The autophagy-targeting chimera degrader LLC355 potently induces lysosomal degradation of DDR1 with a DC50 value of 150.8 nM in lung cancer cells, subsequently inhibiting tumorigenicity, migration, and invasion of cancer cells (101).

Moreover, given the critical role of DDR1 in mediating matrix-dependent drug resistance, a multistage drug delivery system co-loaded with DDR1-siRNA and chemotherapeutic agents has been developed. Small-size siRNA particles could penetrate into tumor cells, silencing DDR1 expression to modulate the collagen-rich tumor matrix, thereby enhancing intratumoral drug permeability and promoting immune cell infiltration (102). Despite these advances, therapeutic agents targeting the full-length DDR1 protein remain to be fully developed and warrant further investigation.

### 4.2 Rational combination therapies with immune checkpoint inhibitors

Through modulation both of extracellular matrix architecture and immune cells, the DDR1 ectodomain orchestrates an immune-excluded microenvironment, thereby fostering tumor progression. Emerging evidence illuminates an unanticipated role for DDR1 in immune evasion, positioning DDR1 as a promising immunotherapeutic target. Given that cancers are mediated by the convergence of multiple dysregulated signaling pathways, multi-target inhibitors or combination therapies present a compelling paradigm for surmounting drug resistance and augmenting therapeutic efficacy. Therefore, combining DDR1 inhibitors with ICIs represents a promising strategy in combinatorial cancer therapy.

DDR1 is frequently upregulated across diverse malignancies, where its overexpression correlates with adverse clinical outcomes. Furthermore, RNA signatures driven by DDR1 kinase activity are tightly linked to resistance against PD-L1 blockade immunotherapy (103). Through an *in vivo* CRISPR/sgRNA screening platform conducted in microsatellite stable (MSS) CRC mouse models, DDR1 was identified as a critical mediator of immunotherapy resistance (55). Notably, combined treatment with dasatinib, a multi-tyrosine kinase inhibitor of both DDR1 and DDR2, and an anti–PD-1 antibody elicited synergistic tumor regression in both MC38 colon cancer and 1956 sarcoma murine models (104). Genetic validation, DDR1-specific knockdown or knockout, is essential to distinguish DDR1-specific effects from broader kinase inhibition. For DDR1-specific evidence, knockdown or inhibition of DDR1 enhanced infiltration of CD8^+^ T cells and the response to PD-1 blockade therapy in MSS CRC but not in microsatellite instability (MSI) CRC (55). Importantly, the therapeutic benefit of DDR1 inhibition was observed specifically in MSS CRC, the subset of colorectal cancers characterized by low mutation burden, poor T cell infiltration, and resistance to immune checkpoint blockade, but not in MSI CRC, which already exhibits high mutation burden and robust T cell infiltration due to neoantigen generation. This differential efficacy highlights that DDR1-mediated immune exclusion is a dominant resistance mechanism in immunologically “cold” MSS tumors, while MSI tumors, which are already immunologically “hot, “ do not rely on DDR1-driven physical exclusion for immune evasion.

Given the compensatory upregulation of DDR2 upon DDR1 inhibition and the reciprocal crosstalk between these receptors, dual DDR1/DDR2 inhibitors may be a promising candidate for combination with ICIs. By simultaneously targeting both receptors, dual inhibitors can more completely disrupt collagen-driven signaling in both epithelial tumor cells and CAFs, potentially overcoming a key resistance mechanism to selective DDR1 monotherapy. This approach aligns with the emerging paradigm that effective immunotherapy combination requires targeting both the tumor cell-intrinsic and stromal compartments of the TIME.

These findings suggest that concurrent targeting of DDR kinases alongside immune checkpoint blockade may represent a promising strategy to potentiate antitumor immunity. This mechanistic insight has propelled PRTH-101 into a Phase 1 clinical trial to evaluate its safety and tolerability alone or in combination with pembrolizumab in patients with advance or metastatic solid tumors.

## 5 Conclusions and perspectives

Functioning as a critical transducer of cancer cells with ECM, DDR1 exerts multifaceted control over cancer biology, tumor dormancy and immunology (12, 105), although the precise molecular mechanisms by which DDR1 orchestrates tumor-immune interactions within the complex tumor microenvironment remain incompletely characterized, particularly regarding its crosstalk with immune effector cells and stromal components.

Given its pivotal role in mediating the complex crosstalk of tumor, stroma and immune cells, DDR1 has emerged as an attractive therapeutic target for concurrently blocking both neoplastic and stromal compartments, especially for TIME modification that may ameliorate response to anti-cancer immune therapy. Despite accumulating evidence implicating the therapeutic effects of DDR1-targeted agents in diverse malignancies, several critical gaps remain unaddressed. First, emerging evidence suggests that DDR1 functions may exhibit substantial heterogeneity across cancer types, necessitating tumor type-specific mechanistic dissection to inform precision therapeutic strategies. Second, the intricate interplay between DDR1 expression dynamics and the functional status of the tumor immune microenvironment remains poorly understood, constraining our ability to fully exploit DDR1 as an immunotherapeutic target. Third, the majority of studies have relied on retrospective analyses and the preclinical models, with prospective clinical validation still limited. Finally, the high structural conservation of the ATP-binding pocket within the DDR1 kinase domain often results in substantial off-target kinase inhibition, which is further compounded by the fact that majority of DDR1 inhibitors frequently exhibit suboptimal selectivity profiles, unacceptable on-target, off-tumor cytotoxicity, and unfavorable drug metabolism and pharmacokinetics properties, collectively hindering their clinical translation.

### 5.1 Context-dependent DDR1 activity reconciles conflicting phenotypes

The apparent contradictions in DDR1 loss-of-function phenotypes across different tumor models can be explained by a context-dependent dual-activity framework that integrates three key layers of regulation.

First, collagen matrix architecture acts as a critical switch. Intact fibrillar collagen binds DDR1 to trigger receptor internalization and degradation, leading to tumor-suppressive outcomes, whereas MMP-cleaved collagen fragments activate a DDR1-NF-κB-p62-NRF2 signaling axis that promotes tumor bioenergetics, macropinocytosis, and metastasis (21). This collagen-quality switch determines whether DDR1 inhibition is beneficial or detrimental, depending on the tumor’s ECM composition and the relative abundance of intact versus cleaved collagen.

Second, cell-type specificity governs the net effect of DDR1 modulation. Intrinsic DDR1 in tumor cells generally promotes malignancy via kinase-dependent signaling through proliferation and metabolic reprogramming, as well as ECD-dependent functions for collagen alignment and immune exclusion. By contrast, DDR1 in stromal fibroblasts and immune cells can exert opposing effects. In CAFs, it regulates matrix stiffness and immunosuppression, whereas in T cells and macrophages it directly modulates migration and polarization. Consequently, the overall effect of DDR1 ablation depends on the relative contributions of these cell-specific activities.

Third, the relative dominance of kinase-dependent versus ECD-dependent signaling varies across tumor subtypes. In cancers where DDR1-mediated immune exclusion primarily depends on the extracellular domain’s collagen-aligning function, kinase inhibition alone may be insufficient. Instead, antibodies targeting the ECD or PROTACs that degrade the full-length receptor may be more effective. Conversely, in tumors where kinase-dependent metabolic reprogramming and proliferation dominate, small-molecule kinase inhibitors may achieve greater therapeutic benefit.

Additional contributing factors, including differences between orthotopic and subcutaneous models, baseline immune composition, treatment timing and dose, and compensatory receptor signaling, are also considered as potential sources of the divergent outcomes. This comprehensive framework for reconciling the apparently contradictory findings in the literature and underscores that DDR1 should be understood as a context-dependent regulator.

### 5.2 Biomarker development and patient stratification

While bulk tumor expression of DDR1 correlates with poor prognosis in retrospective analyses, it is increasingly evident that total receptor levels alone are insufficient to predict therapeutic response. To stratify patients effectively, validated predictive biomarkers are essential. Beyond mere expression level, the phosphorylation status of DDR1 or its immediate downstream effectors might offer a more direct readout of active pathway engagement. Besides, the collagen signature directly modulated by DDR1 activity could identify tumors with “stiff” or “linearized” collagen most reliant on DDR1-directed therapies. Finally, the composition of the immune infiltrate such as a pre-treatment tumor profile characterized by low CD8^+^ T-cell infiltration, high CAF density, and a desmoplastic stroma may represent the patient population most likely to derive benefit from DDR1 inhibition, particularly in combination with ICIs. The development of a composite DDR1-sensitivity signature, integrating gene expression data with histopathological features, is a crucial next step for enabling precision immuno-oncology.

## Glossary

BC: breast cancer
CAF: cancer-associated fibroblast
CML: chronic myeloid leukemia
CRC: colorectal cancer
DC_50_: half-maximal degradation concentration
DDR1: discoidin domain receptor 1
DNMT1: DNA methyltransferase 1
ECs: endothelial cells
ECD: extracellular domain
ECM: extracellular matrix
EGFR: epidermal growth factor receptor
ERK: extracellular signal-regulated kinase
GC: gastric carcinoma
HCC: hepatocellular carcinoma
HSCs: hepatic stellate cells
HUVEC: human umbilical vein endothelial cells
ICIs: immune checkpoint inhibitors
IL-6: interleukin-6
JNK: c-Jun N-terminal kinase
KD: kinase domain
LUAD: lung adenocarcinoma
mAb: monoclonal antibody
MAPK: mitogen-activated protein kinase
MCP-1: monocyte chemoattractant protein-1
MIP-1α: macrophage inflammatory protein-1alpha
MMPs: matrix metalloproteases
MSI: microsatellite instability
MSS: microsatellite stable
NET: neutrophil extracellular trap
NMIIA: non-muscle myosin IIA
NPC: nasopharyngeal carcinoma
NSCLC: non-small cell lung cancer
OSCC: oral squamous cell carcinoma
PDAC: pancreatic ductal adenocarcinoma
PROTACs: proteolysis-targeting chimeras
RTK: receptor tyrosine kinase
Runx2: Runt-related transcription factor 2
SCs: sinusoidal cells
TAMs: tumor-associated macrophages
TIME: tumor immune microenvironment
TME: tumor microenvironment
TNBC: triple-negative breast cancer
VEGF: vascular endothelial growth factor
VSMCs: vascular smooth muscle cells
